# Targeting Neuronal Nitric Oxide Synthase (nNOS) as a Novel Approach to Enhancing the Anti-Melanoma Activity of Immune Checkpoint Inhibitors

**DOI:** 10.3390/pharmaceutics17060691

**Published:** 2025-05-24

**Authors:** Anika Patel, Shirley Tong, Kate Lozada, Amardeep Awasthi, Richard B. Silverman, Jennifer Totonchy, Sun Yang

**Affiliations:** 1 Department of Biomedical and Pharmaceutical Sciences, Harry and Diane Rinker Health Science Campus, Chapman University School of Pharmacy, 9401 Jeronimo Road, Irvine, CA 92618, USA; anikpatel@chapman.edu (A.P.); shirleyftong@gmail.com (S.T.); lozada.kate@gmail.com (K.L.); 2 Department of Chemistry, Northwestern University, Evanston, IL 60208, USA; amardeep.awasthi@northwestern.edu (A.A.); r-silverman@northwestern.edu (R.B.S.); 3 Department of Molecular Biosciences, Chemistry of Life Processes Institute, Center for Developmental Therapeutics, Northwestern University, Evanston, IL 60208, USA; 4 Department of Pharmacy Practice, Harry and Diane Rinker Health Science Campus, Chapman University School of Pharmacy, 9401 Jeronimo Road, Irvine, CA 92618, USA

**Keywords:** melanoma, interferon-gamma (IFN-γ), neuronal nitric oxide synthase (nNOS), nitric oxide (NO), nNOS inhibitors, programmed death-ligand 1 (PD-L1), immune checkpoint inhibitors, peripheral blood mononuclear cells (PBMCs), interleukin-2 (IL-2)

## Abstract

**Background and Objectives**: Neuronal nitric oxide synthase (nNOS) overexpressed in melanoma plays a critical role in disease progression. Our previous studies demonstrated that nNOS inhibitors exhibited potent anti-melanoma activity and regulated PD-L1 expressions in the presence of interferon-gamma (IFN-γ). However, the role of nNOS in the melanoma immune response has not been well defined. **Methods**: Changes in gene expression profiles after nNOS inhibitor treatment were determined by transcriptomic analysis. A melanoma mouse model was used to determine the effects of nNOS inhibition on peripheral T cells and the in vivo anti-tumor activity of combining nNOS inhibitors with immune checkpoint blockade. Changes in human T cell activation through interleukin-2 (IL-2) production were investigated using an ex vivo co-culture system with human melanoma cells. **Results**: Cellular RNA analysis revealed significant changes in the genes involved in key signaling pathways after nNOS inhibitor HH044 treatment. Immunophenotyping of mouse peripheral blood mononuclear cells (PBMCs) after prolonged HH044 treatment showed marked increases in CD4^+^ and CD8^+^PD-1^+^ T cells. Ex vivo studies demonstrated that co-culturing human PBMCs with melanoma cells inhibited T cell activation, decreasing IL-2-secreting T cells both in the presence and absence of IFN-γ. PBMCs from a significant portion of donors (7/11, 64%), however, were reactivated by nNOS inhibitor pretreatment, displaying a significant increase in IL-2^+^ T cells. Distinctive T cell characteristics were noted at baseline among the responders with increased CD4^+^RORγt^+^ and reduced CD4 naïve T cells. In vivo mouse studies demonstrated that nNOS inhibitors, when combined with PD-1 blockade, significantly reduced tumor growth more effectively than monotherapy. Additionally, the median survival was extended from 43 days in the control mice to 176.5 days in mice co-treated with HH044 and anti-PD-1. **Conclusions**: Targeting nNOS is a promising approach to enhancing the anti-melanoma activity of immune checkpoint inhibitors, not only interfering with melanoma biological activities but also regulating the tumor microenvironment, which subsequently affects T cell activation and tumor immune response.

## 1. Introduction

Cutaneous melanoma (CM) incidence rates have continued to increase in recent decades, making this disease a rising public health concern in the United States [1]. Melanoma accounts for less than 1% of all skin cancer cases but the most skin cancer deaths, and it is highly resistant to classical chemotherapy [2]. Early diagnosis and excision of the localized cancerous tissue are primarily curative. However, once this disease becomes metastatic, the 5-year survival rate drops to 35% [3]. The breakthrough development of targeted therapies and immunotherapies has completely changed the landscape of melanoma treatment over the last two decades. Nevertheless, metastatic melanoma remains the deadliest and most aggressive form of skin cancer [4].

Immune checkpoint inhibitors (ICIs) blocking cytotoxic T-lymphocyte associated protein 4 (CTLA-4) and programmed cell death protein 1 (PD-1) have shown promising clinical efficacy in patients diagnosed with melanoma [5]. Although anti-PD-1 agents significantly improved the overall response rate (ORR) and overall survival (OS) compared with traditional chemotherapy, a significant population of patients fail to respond to immunotherapy or develop resistance [6]. The ORR and OS of anti-PD-1 therapy can be improved when used in combination with anti-CTLA-4 ICIs. However, combination therapy results in a significantly increased prevalence of toxicities [7]. Additionally, despite promising anti-melanoma activity, immunotherapy faces its own challenges, such as a lack of predictive biomarkers for patient response and risk of severe immune-related adverse events (irAEs) [8]. Often, immunotherapy is discontinued due to severe irAEs, which may manifest in the skin, gastrointestinal tract, liver, and endocrine organs [9,10]. However, immunotherapy is still projected to hold the largest market share within the therapy segment by 2030 due to its proven efficacy and increasing number of FDA approvals [11]. A robust emerging pipeline of ICIs is expected to drive melanoma drug development over the next five years. Research focused on developing novel strategies to enhance immunotherapy has also gained increasing interest in the field. In recent years, several combinations of anti-PD-1 and other therapies are being studied for melanoma, including combinations with interleukin-2 conjugates, oncolytic viral therapy, anti-lymphocyte activation gene (LAG)-3 antibodies, and IDO1 inhibitors [12]. Additionally, patient-derived T cell therapies are currently being explored as more personalized immunotherapy, aligning with the recent FDA approval of Amtagvi [13].

Neuronal nitric oxide synthase (nNOS), expressed primarily in neuronal tissue, plays a prominent role in regulating nitric oxide (NO) levels in melanocytes [14]. Our previous studies showed that nNOS, which is markedly overexpressed in CM, is a promising druggable target for melanoma treatment [15,16]. Small molecule inhibitors selectively inhibiting the enzymic activity of nNOS exhibited potent anti-melanoma activities both in vitro and in vivo [17]. An earlier study by Liu et al. showed that elevated nNOS expression in human melanoma tissue was linked to immune dysfunction in circulating lymphocytes, resulting in immunosuppression [18]. Consistently, other studies showed that the dynamic regulation of NO was associated with immunosuppression in many types of cancers [19,20], which may contribute to UV-induced changes in immune activity in the skin [21]. As a result, NO stimulates cell proliferation and facilitates cancer invasion observed in melanoma and non-melanoma malignancies [22,23,24]. Thus, nNOS inhibitors may hinder NO-mediated immune regulation, cancer cell proliferation, and invasion [15,19,25].

Our previous studies showed that nNOS inhibitors reduced the expression of programmed death-ligand 1 (PD-L1) in the presence of interferon-gamma (IFN-γ), which is a known pro-tumorigenic cytokine stimulating melanoma progression observed in earlier clinical trials and animal studies [16,17,26,27]. Recently, our group further demonstrated that crosstalk between the nNOS/NO and COX-2/PGE_2_ signaling pathways amplified IFN-γ-stimulated PD-L1 expression in melanoma cells [16]. These observations suggest that nNOS-mediated NO signaling may impact the tumor immune microenvironment and regulate PD-L1-mediated immune suppression [17]. We hypothesized that targeting nNOS may enhance or potentiate the anti-melanoma effects of anti-PD-1 ICIs by inhibiting PD-L1 expression in melanoma cells. In this study, we aim to determine the in vivo anti-tumor efficacy of utilizing nNOS inhibitors as adjuvant treatment with ICIs and further define the role of nNOS inhibition on the immune profiles and T cell activation.

## 2. Materials and Methods

### 2.1. Cell Lines, Chemicals, and Reagents

Human metastatic melanoma A375 and murine melanoma Cloudman S91 (CS91) cells were obtained from the American Type Culture Collection (ATCC; Manassas, VA, USA). A375 cells were cultured in Dulbecco’s Modified Eagle’s Medium (DMEM; #11995073; Gibco, Waltham, MA, USA) with 10% fetal bovine serum (FBS; #26140079; Gibco, Waltham, MA, USA). CS91 cells were cultured in F-12K Medium (#30-2004; ATCC) supplemented with 2.5% FBS and 15% horse serum (HS; #30-2040; ATCC). The human immortal melanocyte cell line Hermes 1 was generously provided by Professor Dorothy C. Bennett (University of London, London, UK). The culture media and conditions followed the directions provided by the Functional Genomics Cell Bank at St. George’s University of London [28].

Anti-mouse PD-1 (clone RMP1-14) antibody (ICH1132) and rat IgG2a isotype control (ICH2244) were purchased from Ichorbio (Wantage, UK) for the in vivo mouse studies. IFN-γ was supplied by GoldBio (#1160-06-100; St. Louis, MO, USA). Phorbol 12-myristate 13-acetate (PMA), ionomycin, and monensin were supplied by Sigma-Aldrich (St. Louis, MO, USA), and primocin was supplied by InvivoGen (San Diego, CA, USA).

### 2.2. Novel nNOS Inhibitors

The nNOS inhibitors (HH044 and MAC-3-190; purity > 99%, determined by HPLC) were synthesized by the Silverman group at Northwestern University (Evanston, IL, USA), and nNOS inhibitory and selectivity assays were conducted as described previously [29,30]. The *K*_i_ values were calculated from the IC_50_ values of the corresponding dose-response curves using the Cheng-Prusoff equation. For each compound, 8–11 concentrations were tested, and the IC_50_ value was calculated from an average of at least two duplicates. All standard errors were less than 5%. Selectivity values were determined by calculating the ratios of the respective *K*_i_ values.

### 2.3. Nanostring Gene Expression Profiling

After treatment, the total RNA samples were collected from A375 cells using an RNeasy Mini Kit (#74104; Qiagen, Hilden, Germany) and stored at −80 °C prior to analysis. The nCounter PanCancer IO 360 Panel and nCounter Pro Analysis System (NanoString, Seattle, WA, USA) were used to quantify gene expression changes in 770 genes implicated in immune-oncological processes. The transcriptomic data were analyzed using ROSALIND online platform (https://www.rosalind.bio/, San Diego, CA, USA).

### 2.4. In Vivo Immunocompetent Allograft Melanoma Mouse Model for Immune Profile Analysis of Peripheral Blood Mononuclear Cells (PBMCs)

The in vivo studies were carried out in compliance with the ARRIVE guidelines. All animal procedures were approved by the Institutional Animal Care and Use Committee (IACUC) at Chapman University and conducted in compliance with the policies of Chapman University and federal, state, and local animal welfare authorities. DBA/2 mice were purchased from Charles River (Wilmington, MA, USA) and housed in the Chapman University vivarium. Male mice were selected for the in vivo studies due to the markedly slow growth rate of CS91 cells in female mice observed in previous studies [31]. To induce melanoma tumor growth, the mice were injected subcutaneously with 1 × 10^5^ CS91 murine melanoma cells in a 100 μL solution of 50% Matrigel basement membrane matrix (CB354248, Corning, Corning, NY, USA). The mice were randomized into the control vehicle (0.9% sodium chloride) or HH044 10 mg/kg/day intraperitoneal treatment groups and treated for 26 days. At the end of the study, peripheral blood was collected from the mice for T cell analysis.

### 2.5. T Cell Staining for Flow Cytometry Analysis

Cells were blocked in FACS block buffer (0.5% BSA, 2% FBS in 1× PBS) and incubated in a surface marker antibody dilution in FACS wash buffer (0.5% BSA, 0.05% NaN_3_ in 1× PBS). Following fixation with True-Nuclear Fix Buffer (424401; BioLegend, San Diego, CA, USA), the cells were incubated with an intracellular antibody diluted in True-Nuclear Perm Buffer (424401; BioLegend, San Diego, CA, USA) overnight at 4 °C. The stained cells were then washed with Perm Buffer and analyzed using a BD Fortessa X20 flow cytometer (BD Biosciences, San Jose, CA, USA). Compensation beads (anti-mouse 552843; anti-rat 552844; BD Biosciences, Franklin Lakes, NJ, USA) stained with single antibodies were used to calculate the compensation, and data were analyzed and gated using FlowJo v. 10.8.1 (FlowJo LLC, Ashland, OR, USA).

### 2.6. Analysis of Mouse PBMCs Using Flow Cytometry

Peripheral blood samples were collected from the DBA/2 mice at the end of the study. T cells were isolated from the blood using Ficoll separation and stained as described above [32]. The Ficoll-Paque solution was obtained from Cytiva (17144002; Marlborough, MA, USA). The T cell marker antibodies used were Brilliant Violet 605 anti-mouse CD3, PE/Cyanine7 anti-mouse CD4, Brilliant Violet 785 anti-mouse CD8a, APC/Cyanine7 anti-mouse CD279 (PD-1), Alexa Fluor 647 anti-GATA3, Alexa Fluor 488 anti-mouse FOXP3, Brilliant Violet 421 anti-T-bet (100237; 100421; 100749; 135223; 653809; 126406; 644815; BioLegend, San Diego, CA, USA), and PE-CF594 mouse anti-mouse RORγt (562684; BD Biosciences, Franklin Lakes, NJ, USA).

### 2.7. Isolation of Human PBMCs from Healthy Donors and Collection of CD3^+^ T Cells

Freshly collected peripheral blood samples from healthy donors between 18 and 50 years old were purchased commercially (HUMANWBK2UZN; BioIVT; Hicksville, NY, USA) [33]. The blood samples were tested using FDA CBER licensed screening assays and confirmed to be free of infectious agents prior to shipment. PBMCs were isolated by a density gradient using Ficoll, cryopreserved in 10% DMSO in FBS, and stored in liquid nitrogen. To further isolate the T cells, the PBMCs were rapidly thawed and diluted dropwise in 10 mL RPMI1640 supplemented with 20% FBS, 50 mg/mL primocin, and 10 µg/mL DNase 1. The cells were allowed to recover for 2 h in the incubator before proceeding with CD3^+^ T cell isolation using MojoSort magnet sorting (BD Biosciences).

Cells were filtered through a 20 µm pre-separation filter (Miltenyi Biotec, San Diego, CA, USA) to remove dead and aggregated cells and then washed with 1× MojoSort buffer. The cell suspension (100 µL, 10^7^ cells/mL) was aliquoted, and 10 µL of Biotin-Antibody Cocktail was added and incubated for 15 min. Streptavidin nanobeads (10 µL) were added to the cell suspension. After incubation for 15 min, MojoSort buffer was added and incubated inside the MojoSort magnet for 5 min. Leaving the tube inside the magnet, the supernatant was collected into a separate tube containing the isolated CD3^+^ T cells. The cells were then washed via centrifugation and resuspended in RPMI 1640 supplemented medium. The cells recovered overnight in the incubator before co-culturing with melanoma cells.

### 2.8. Ex Vivo Co-Culture of Human Melanoma and CD3^+^ T Lymphocytes

The human melanoma A375 cells (1 × 10^5^) were pretreated with IFN-γ (250 units/mL) and MAC-3-190 in serum-free media for 48 h. For co-culturing, 1 × 10^6^ CD3^+^ T cells in 1 mL RPMI 1640 supplemented medium were added directly to the pretreated melanoma cells and incubated for 24 h. The cells were then stimulated for 3 h with PMA (10 ng/mL) and ionomycin (500 ng/mL), followed by treatment with monensin (1 µM) for 3 h to inhibit cytokine secretion and facilitate intracellular cytokine accumulation for quantitative detection. The T cells were then collected and stained for flow cytometry analysis.

### 2.9. Flow Cytometry Analysis of Human PBMC Cells

Flow cytometry was used to first detect viable CD3^+^, CD4^+^, CD8^+^, and interleukin-2 (IL-2) positive T cells from human PBMCs. The T cell marker antibodies used for this analysis were anti-human CD3 PE (BD; #561809), anti-human CD4 FITC (BD #557695), anti-human CD8 APC-Cy7 (BD; #560179), and anti-human IL-2 V450 (BD; #562914). The T cells were stained as described above and then fixed with 4% paraformaldehyde for 10 min at ambient temperature in the dark. The cells were permeabilized with saponin for 15 min prior to incubation with the intracellular antibody dilution. Data were acquired on the BD FACS Verse flow cytometer.

### 2.10. Profiling of Human PBMCs Using a CD Marker Panel

The human T cell surface marker antibodies used in this study were anti-human CD8-BUV496 (BD; #612942), anti-human CD25-BUV737 (BD; #612807), anti-human CD45RO-FITC (BD; #555392), anti-human CD19-BV510 (BD; #562953), anti-human CD45RA-BV650 (BioLegend; #304136), anti-human PD-1-BV786 (BD; #329930), anti-human CCR7-PE (BD; #566742), anti-human CD95-PE-Cy5 (BioLegend; #305610), anti-human CD28-PE-Cy7 (BD; #302925), and anti-human CXCR5-Alexa Fluor 700 (BioLegend; #356916), anti-human CD4-APC-Cy7 (BD; #560158), and the intracellular cytokine and transcription factor antibodies were anti-human IFN-γ-BUV395 (BD; #563563), anti-human RORγt-BV421 (BD; #563282), anti-human BCL6-BV711 (BD; #567180), and anti-human FOXp3-BB700 (BD; #566527).

At 24 h post-thaw, 1 × 10^6^ PBMCs were aliquoted and stained with viability stain BV510 on ice for 15 min in the dark. The cells were blocked in FACS block buffer and incubated in a surface marker antibody dilution in FACS wash buffer containing 10 µL BD Brilliant Stain Buffer Plus (BD; #566385) for 15 min on ice protected from light. After the cells were fixed with True-Nuclear Fix buffer for 10 min at ambient temperature in the dark, they were permeabilized with saponin for 15 min. The cells were then incubated in an intracellular antibody dilution in True-Nuclear Perm Buffer containing 10 µL BD Brilliant Stain Buffer Plus for 15 min at ambient temperature in the dark. The stained cells were then analyzed via flow cytometry on a BD Fortessa X20 flow cytometer.

### 2.11. In Vivo Anti-Melanoma Efficacy of nNOS Inhibitors in Combination with ICIs Using an Immunocompetent Allograft Melanoma Mouse Model

As described above, male DBA/2 mice were purchased from Charles River and housed in the Chapman University vivarium under pathogen-free conditions. Each mouse received a subcutaneous injection in the flank with 0.25 × 10^6^ CS91 cells suspended in 50% Matrigel. The mice were randomized for treatment and were given intraperitoneal injections of either the control (rat IgG2a isotype control twice per week for 2 weeks, 0.9% sodium chloride, daily), HH044 (10 mg/kg/day), MAC-3-190 (10 mg/kg/day), anti-PD-1 (100 µg twice per week for 2 weeks), or a combination of the nNOS inhibitor and ICI treatment, starting from 3 days after tumor inoculation. The same dose was used for HH044 and MAC-3-190 to facilitate a direct comparison of their anti-tumor efficacy. The body weight and tumor volume were measured twice weekly for three weeks. Tumors were measured using calipers, and the volume (mm^3^) was calculated with the following formula: volume = [length × (width^2^)]/2. The mice were sacrificed once their tumors reached 2000 mm^3^, and dates were recorded for survival analysis.

### 2.12. Ex Vivo Sensitivity of Murine Melanoma Cells to nNOS Inhibitors After Prolonged Treatment

Tumor allografts were harvested from the mice following ≥21 days of MAC-3-190 or HH044 treatment (intraperitoneal, 10 mg/kg/day). The aseptically collected tumor samples were mechanically dissociated in 1× PBS and passed through a 40 µm cell strainer. Single cell suspensions were centrifuged and resuspended in full growth media supplemented with 100 µg/mL of primocin. The cells were then plated and allowed to adhere for 24 h. Afterward, the cultures were washed twice with 1× PBS to remove non-adherent cells and allowed to grow in full growth media.

The viability of the cells in response to MAC-3-190 and HH044 treatment was determined using MTT colorimetric analysis as previously described [34]. GraphPad Prism 9 was used to determine the IC_50_ of each compound by plotting the percent cell viability against the log drug concentrations and fitted using a nonlinear fit of the normalized data.

### 2.13. Statistical Analysis

Statistical analyses were carried out using GraphPad Prism 9. Comparisons of multiple groups were performed using mixed-effects analysis of variance (ANOVA) followed by Tukey’s post hoc test for parametric data. Statistical comparisons of two groups were performed using an unpaired two-tailed *t*-test for parametric data. Statistical comparison for a simple 2 × 2 contingency table was performed using a Chi-Square calculator on Social Science Statistics. Kaplan Meier methods were utilized for estimating the survival probability over time, with a tumor volume of 2000 mm^3^ marking the endpoint for the survival study. A *p* value less than 0.05 was considered statistically significant for all statistical analyses. Data are graphically represented as bar graphs with the mean ± standard error of the mean (SEM).

## 3. Results

### 3.1. Novel Synthesized Selective nNOS Inhibitors

Targeting nNOS shows great promise in inhibiting human melanoma progression and tumor growth [15,17,35]. However, the crystal structures of the oxygenase domains of NOS enzymes indicate a rather high degree of structural similarity within the catalytic center and dimer interface regions among the isoforms [36,37,38,39,40,41,42]. To avoid the detrimental effects of iNOS and eNOS inhibition noted above, highly selective nNOS inhibition is essential for melanoma therapy. Our interdisciplinary team, led by the Silverman group, has successfully elucidated the structural basis for a group of highly selective nNOS inhibitors. HH044 and MAC-3-190 have been identified as our lead compounds, which exhibited selective nNOS inhibition and potent anti-melanoma activities in vivo [42,43,44,45,46,47]. As shown in Table 1, the selectivity of HH044 for nNOS inhibition was 337-fold and 61-fold over eNOS and iNOS, respectively. MAC-3-190 exhibited potent nNOS inhibition (*K*_i_ hnNOS = 0.033 μM), being 119-fold and 89-fold more selective over eNOS and iNOS, respectively.

### 3.2. The nNOS Inhibitor Changed the Gene Expression Characteristics of Human Melanoma A375 Cells

**Figure 1:**
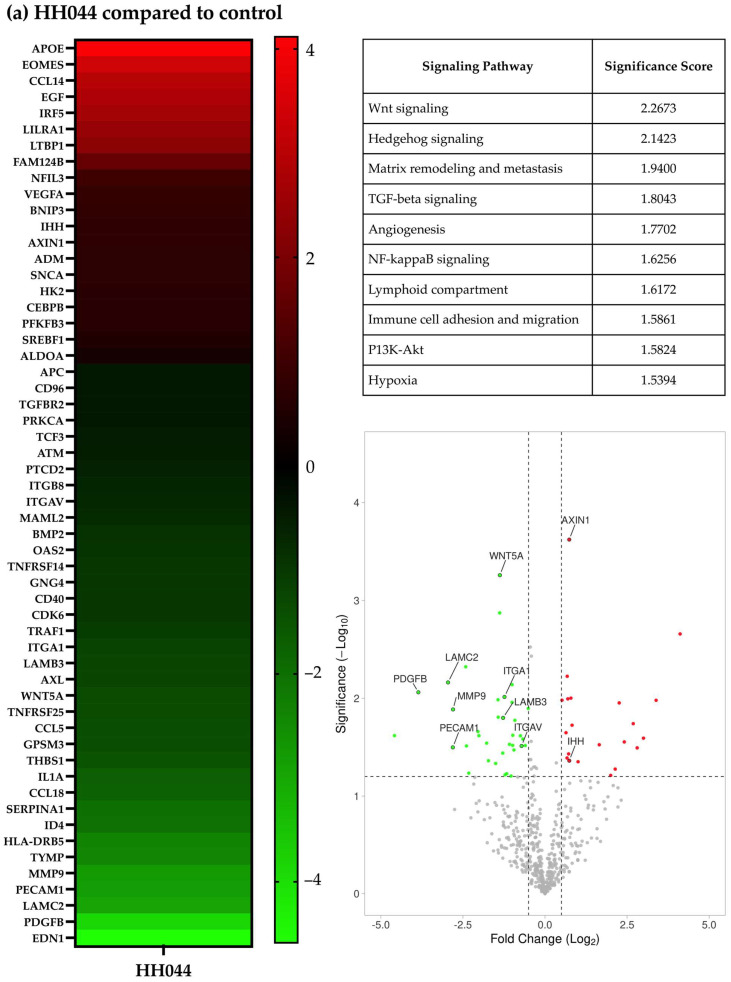

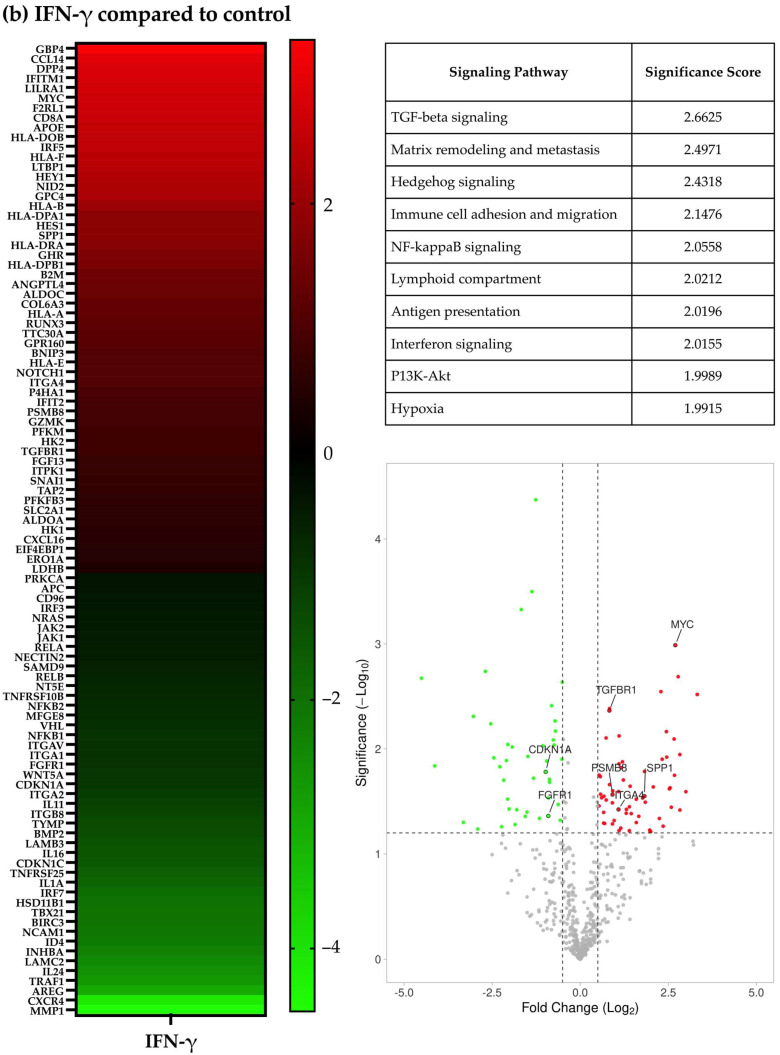

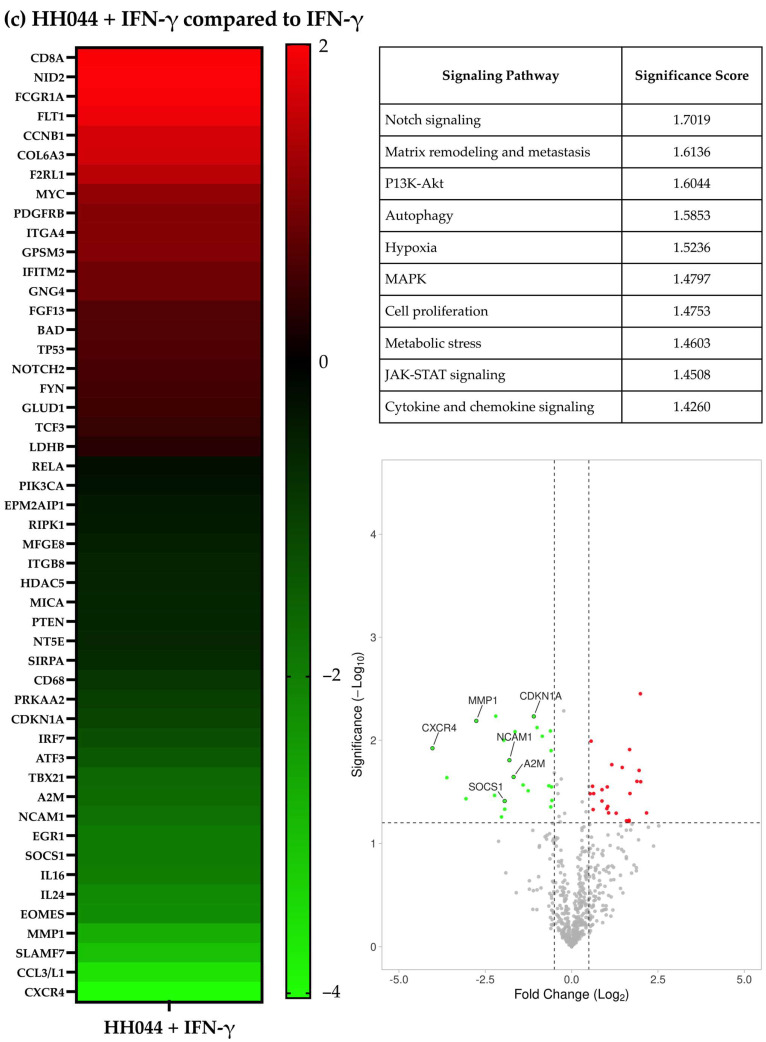
Gene expression characteristics of human melanoma cells treated with HH044 in the presence and absence of IFN-γ. The nCounter PanCancer IO 360 Panel was used to analyze changes in the gene expression characteristics after treatment with HH044 (20 μM), IFN-γ (250 units/mL), or a combination treatment for 54 h. (**a**) Comparing HH044 treatment to control. (**b**) Comparing IFN-γ treatment to control. (**c**) Comparing the combination treatment to IFN-γ alone. The heatmaps show the top differentially expressed genes predictive of treatment based on log2 (fold change). All the treatments were independently repeated three times. The expression of the listed genes was statistically significantly changed (*p* < 0.05). The tables list the biological pathways that were significantly impacted by the treatment. The analysis was based on the expression changes of genes involved in each specific pathway, with a higher score signifying a greater probability of pathway involvement. The volcano plots showed the genes significantly upregulated (red) or downregulated (green) by treatment (*p* < 0.05). Labeled genes were implicated in the affected biological pathways.

The A375 cells were treated with HH044 for 54 h before significant cell death was observed. Noteworthy changes in the expression of various cancers and immunoregulatory genes were detected by NanoString nCounter analysis using a PanCancer IO 360 Panel. In comparison with the control, 18 genes were statistically significantly upregulated, and 30 genes were downregulated (Figure 1a heatmap; *p* < 0.05). Of note, HH044 treatment significantly reduced the expression of EDN1, PDGFB, and MMP9, which are known to facilitate melanoma disease progression and serve as druggable targets for pharmacotherapy [48,49,50,51,52,53,54,55].

Based on the observed gene expression changes, the prominently implicated signaling pathways included the Wnt signaling, Hedgehog signaling, TGF-beta, NF-kappaB, and PI3K-Akt pathways. Additionally, signaling pathways associated with matrix remodeling and metastasis, lymphoid compartment, and immune cell adhesion and migration were also notably altered by HH044 treatment (Figure 1a table). Some of these genes are highlighted in the volcano plot in Figure 1a. The full list of implicated pathways and associated changes in gene expression is presented in Supplementary Table S1. IL-1α expression was reduced to 30.3% of the control (*p* < 0.05). Earlier studies showed IL-1α, a cytokine that can orchestrate an inflammatory response in the tumor microenvironment, mediates the innate and acquired resistance to immunotherapy in melanoma cells [56,57,58,59]. Transcriptomic analysis suggests that inhibition of nNOS not only directly interferes with melanoma growth but may also impact the immune response within the tumor microenvironment.

When comparing the IFN-γ treatment to the control, 47 genes were upregulated, and 36 were downregulated (Figure 1b). The three signaling pathways most significantly altered by IFN-γ were TGF-beta signaling, matrix remodeling and metastasis, and Hedgehog signaling (Figure 1b and Supplementary Table S2).

The gene expression changes with HH044 cotreatment in the presence of IFN-γ were also analyzed. In comparison with IFN-γ alone, 16 genes were upregulated, and 21 genes were downregulated significantly (*p* < 0.05, Figure 1c and Supplementary Table S3). These gene changes presented as noteworthy in the context of Notch signaling, matrix remodeling and metastasis, and P13K-Akt signaling (Figure 1c). Certain genes involved in the implicated signaling pathways are noted in the volcano plots in Figure 1c.

### 3.3. The nNOS Inhibitor Treatment Altered the Peripheral T Cell Profile in Mice

**Figure 2:**
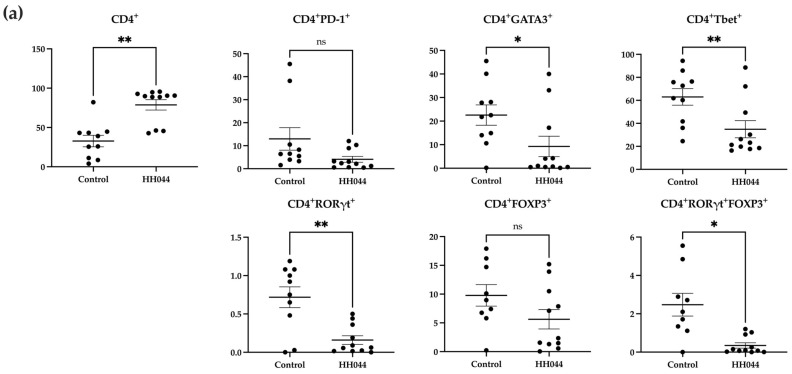

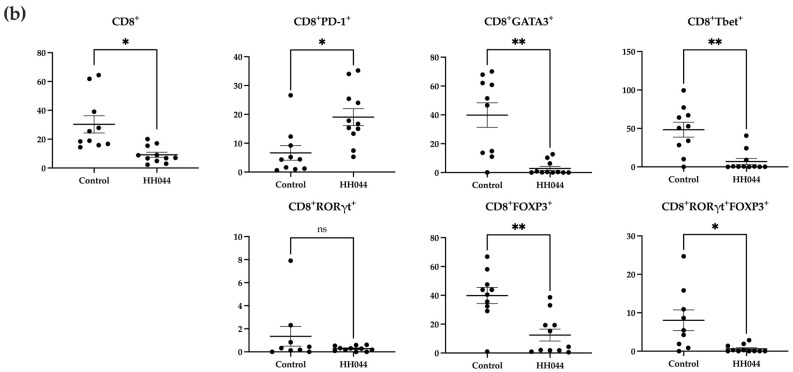
Alterations in peripheral T cell subsets following nNOS inhibitor treatment in mice. Percentages are representative of T cells out of the total number of viable (**a**) CD4^+^ and (**b**) CD8^+^ T cells detected using flow cytometry. PBMCs were isolated from mouse peripheral blood after 24 days of treatment with the control vehicle (*n* = 9) or HH044 (10 mg/kg, intraperitoneal, daily, n = 11). Statistical analysis was computed using unpaired *t*-tests, and error bars denote mean ± SEM (* *p* < 0.05; ** *p* < 0.01; ns = non-significant). Outliers were detected and removed using Grubbs’ test (α = 0.05).

Flow cytometry analysis showed notable changes in the percentages of various CD4^+^ and CD8^+^ T cell subtypes from the peripheral blood after treatment (Figure 2). The gating strategy for this analysis is shown in Supplementary Figure S1.

The mice treated with HH044 demonstrated significantly increased CD4^+^ T cell counts compared with the control group (Figure 2a). However, within this population, the nNOS inhibitor treatment decreased CD4^+^GATA3^+^, CD4^+^RORγt^+^, CD4^+^Tbet^+^, and CD4^+^RORγt^+^FOXP3^+^ T cell counts markedly.

The overall percentage of CD8^+^ T cells was significantly decreased in addition to specific CD8^+^ subsets, including CD8^+^FOXP3^+^, CD8^+^GATA3^+^, CD8^+^Tbet^+^, and CD8^+^RORγt^+^ FOXP3^+^ double positive T cells (Figure 2b). In contrast, the frequency of CD8^+^PD-1^+^ T cells markedly increased after treatment.

### 3.4. Co-Treatment with nNOS Inhibitor Increased Human IL-2^+^ T Cells in the Absence and Presence of IFN-γ Ex Vivo

**Figure 3:**
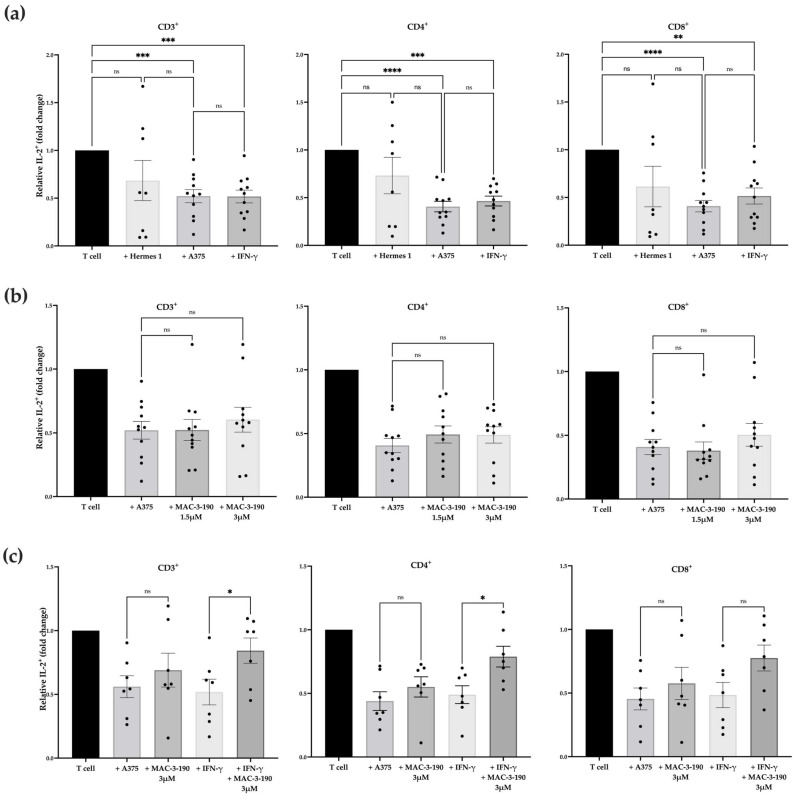
Changes in human IL-2^+^ T cells co-cultured with melanoma cells. Flow cytometric analyses depicting the percentage of IL-2^+^ T cells normalized to T cells stimulated with PMA and ionomycin. (**a**) The percentage of IL-2^+^ T cells significantly decreased following ex vivo co-culture with A375 cells with or without IFN-γ-pretreatment compared to PMA-stimulated T cells alone (n = 11). A non-significant decrease in IL-2^+^ T cells was observed with Hermes 1 co-incubation (n = 8). (**b**) The percentage of IL-2^+^ CD3^+^, CD4^+^, and CD8^+^ T cells increased slightly after treatment with 3.0 µM MAC-3-190 compared with T cells co-incubated with A375 cells (n = 11). (**c**) The proportion of IL-2^+^ cells within the CD3^+^ and CD4^+^ T cell populations significantly increased following co-culture with A375 cells pretreated with IFN-γ and MAC-3-190 compared with cells pretreated with IFN-γ only among the responsive donors (*p* < 0.05; n = 7). Statistical analysis was computed using mixed-effects ANOVA, and error bars denote mean ± SEM (* *p* < 0.05; ** *p* < 0.005; *** *p* < 0.001; **** *p* < 0.0001; ns, non-significant).

To evaluate the effects of nNOS blockade in melanoma on human T cell activation, we utilized an ex vivo co-culture model with human melanoma cells and PBMCs isolated from healthy donors. We first evaluated T cell activation via intracellular cytokine staining for IL-2 after coincubation with A375 with and without IFN-γ pretreatment (250 units/mL for 48 h). The gating strategy for this analysis is shown in Supplementary Figure S2. As shown in Figure 3a, the frequency of IL-2^+^ cells within CD3^+^, CD4^+^, and CD8^+^ T cell populations significantly decreased after coincubation with human melanoma A375 cells regardless of IFN-γ pre-exposure (*p* < 0.05), while no significant change was seen after coincubation with Hermes 1 cells (Supplementary Figure S3).

Next, the A375 cells were pretreated with nNOS inhibitor MAC-3-190 (1.5 µM or 3.0 µM) in the presence or absence of IFN-γ (250 units/mL) for 48 h, followed by coincubation with CD3^+^ T cells. As shown in Figure 3b, pretreatment with MAC-3-190 alone did not significantly increase the IL-2^+^ T cell count compared with the control T cells after coincubation with melanoma cells. However, in the presence of IFN-γ, MAC-3-190 pretreatment exhibited a non-significant trend toward an increase in IL-2^+^ T cells with increasing doses from 1.5 µM to 3 µM (Supplementary Figure S4). Similar trends were observed in CD4^+^IL-2^+^ T cells with MAC-3-190 co-treatment with IFN-γ (increasing from 0.47-fold to 0.60-fold for T cells alone). Due to the small sample size of donors (n = 11), the increases observed were not statistically significant.

Further analysis demonstrated that PBMC-derived T cells collected from seven donors were identified as responsive to MAC-3-190 treatment from the 11 total donors, who exhibited a significant increase in IL-2^+^ cells in CD3^+^ and CD4^+^ cell populations after pretreatment with MAC-3-190 (3 µM) for 48 h with IFN-γ, compared with the cells exposed to IFN-γ alone (Figure 3c; *p* < 0.05). Increased IL-2^+^ T cells were also evident in the CD8^+^ populations. However, such induction after cotreatment was not statistically significant compared with IFN-γ alone. A representative dot plot (Supplementary Figure S5) shows an increase in IL-2^+^ cells after nNOS inhibitor treatment with and without IFN-γ in Donor 03. Of these seven responders, 85.7% (6/7) were male patients. Based on the self-reported ethnicities, 85.7% (6/7) were Caucasian, and 14.5% (1/7) were African American. One responder reported red hair, and one was unknown, while the majority were blonde (71.4%, 5/7). Among these seven donors, 28.5% were between the ages of 20 and 29, 42.9% were between the ages of 30 and 39, and 28.5% were between the ages of 40 and 49.

### 3.5. Naïve CD4^lo^ and CD4^+^RORγt^hi^ T Cells Present in Donors Responding to nNOS Inhibitor Treatment

**Figure 4:**
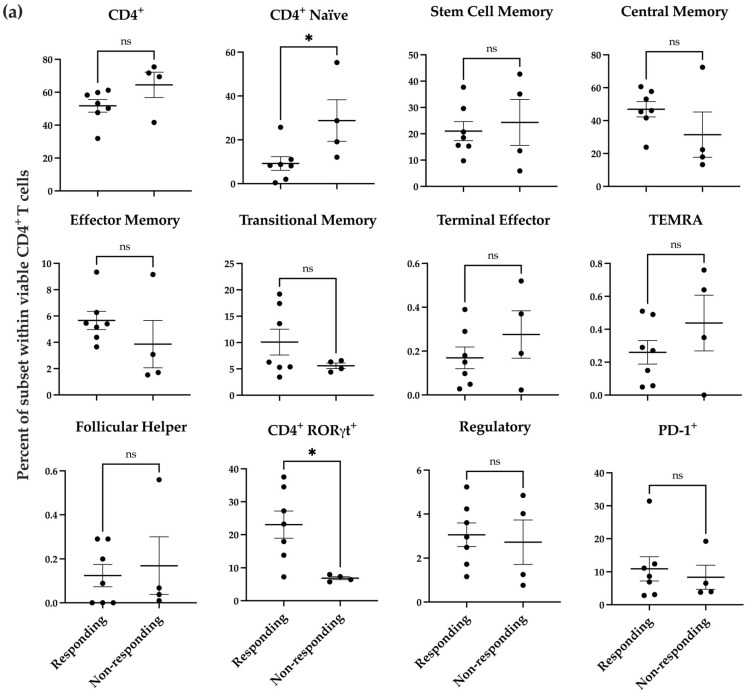

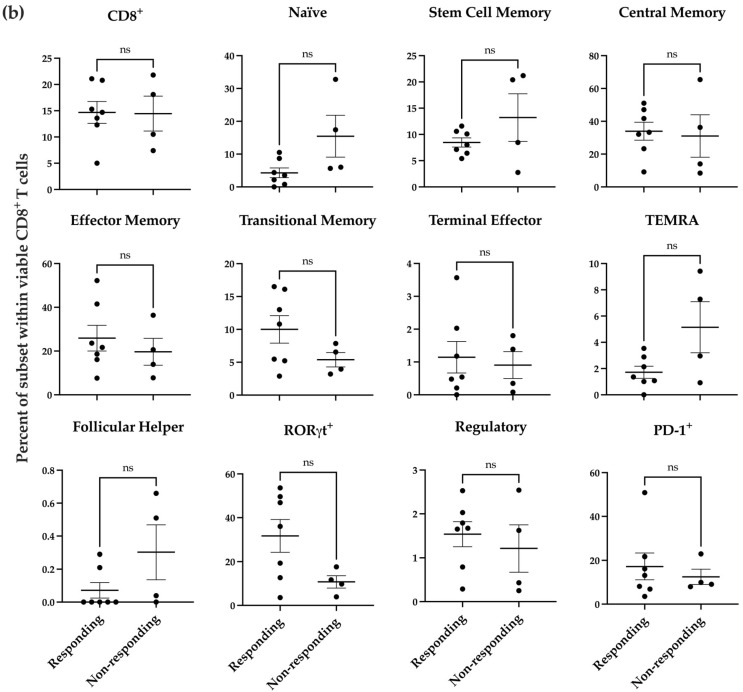
Baseline profile of donor PBMC-derived T cell subsets prior to coincubation with melanoma cells. Distribution of T cell subsets within viable CD4^+^ (**a**) and CD8^+^ (**b**) T cells in responding donors (n = 7) compared to non-responding donors (n = 4). Statistical analysis was computed using unpaired *t*-tests, and error bars denote mean ± SEM (* *p* < 0.05; ns = non-significant).

To determine if the donor-specific response we observed in the melanoma co-culture after nNOS inhibitor treatment was associated with preexisting immunological states in the donor samples, we used a more detailed T cell immunophenotyping panel (Supplementary Figure S6) to profile the T cell populations in the donor samples. T cell subsets are shown for the CD4^+^ cells (Figure 4a) and CD8^+^ cells (Figure 4b) separately. These results reveal that there were significant differences in the percent of CD4^+^ naïve and CD4^+^ RORγt^+^ T cells between responding and non-responding donors (Figure 4a, *p* < 0.05).

### 3.6. Co-Treatment of nNOS Inhibitors Enhanced the Anti-Tumor Efficacy of Anti-PD-1 Immunotherapy

To determine whether nNOS blockade enhanced the anti-melanoma activity of immune checkpoint inhibitors, we used an immunocompetent murine allograft tumor melanoma model, which has been used in preclinical studies and shown to be responsive to immunotherapy, mimicking patient responses [31,60]. The in vivo treatment dosing regimen was chosen based on our previous mouse studies, which showed promising anti-melanoma activity without severe systemic toxicities [17].

**Figure 5:**
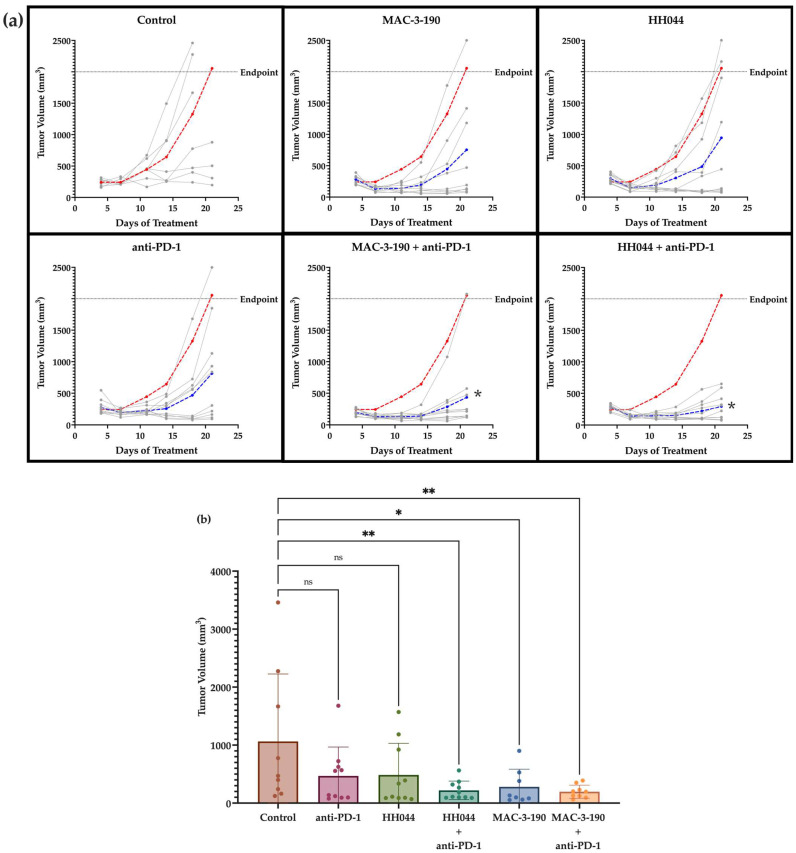
Anti-melanoma activity of nNOS inhibitors alone and in combination with anti-PD-1 immune checkpoint blockade. Murine melanoma CS91 cells were injected into DBA/2 mice subcutaneously on the flank. The control mice were given intraperitoneal injections of a saline vehicle (daily) and rat IgG2a isotype control (twice per week for 2 weeks) (n = 10). Treatment groups included HH044 (10 mg/kg/day), MAC-3-190 (10 mg/kg/day), anti-PD-1 (100 µg, twice per week for 2 weeks), and the combination of an nNOS inhibitor and immunotherapy (n = 10 per group). (**a**) All treatment groups (blue) exhibited slower tumor growth rates compared with the control (red), shown as the average tumor volume over the course of 21 days of treatment. Individual tumor growth curves are represented in gray. The combination of anti-PD-1 immunotherapy with nNOS blockade showed significant differences in tumor growth compared with the control, as determined by two-way ANOVA (* *p* < 0.05 compared with control). (**b**) Effect of nNOS inhibitors alone and in combination with anti-PD-1 on average tumor volume by day 18 of treatment (* *p* < 0.05; ** *p* < 0.005 compared with control; ns, non-significant). Statistical analysis was performed with one-way ANOVA. Outliers were detected and removed using Grubbs’ test (α = 0.05).

Figure 5a shows the tumor growth curves of the individual mice in each group. Of note, in the combination treatment groups (HH044 + anti-PD-1 and MAC-3-190 + anti-PD-1), the tumor growth was significantly slower compared with the control mice, and the responses for each treatment within the same group were consistent. As shown in Figure 5b, on day 18, HH044 and MAC-3-190 treatments alone reduced tumor growth to 45.7% and 26.3% of the control, respectively, while anti-PD-1 monotherapy reduced the tumor volume to 44.0% of the control group. MAC-3-190 demonstrated slightly better in vivo anti-melanoma activity at the same dosage (10 mg/kg/day, intraperitoneal, daily) compared with HH044.

By day 18, co-treatment of anti-PD-1 with nNOS inhibitors further inhibited tumor growth and significantly reduced the average tumor volume. Of note, the combination of HH044 with anti-PD-1 inhibited tumor growth to 20.7% of the control group (Figure 5b). MAC-3-190 co-treatment also markedly enhanced the anti-melanoma activity of anti-PD-1 by reducing the average tumor volume to 18.5% of the control.

With prolonged treatment, the mice treated with MAC-3-190 or anti-PD-1 therapy did not exhibit significant changes in body weight. However, the body weights of the mice treated for 21 days with HH044 alone and in combination with anti-PD-1 therapy displayed significantly lower mean body weights compared with the control (Supplementary Figure S7a).

### 3.7. Co-Treatment with nNOS Inhibitor HH044 and Anti-PD-1 Immunotherapy Significantly Extended Survival in the Murine Melanoma Model

**Figure 6:**
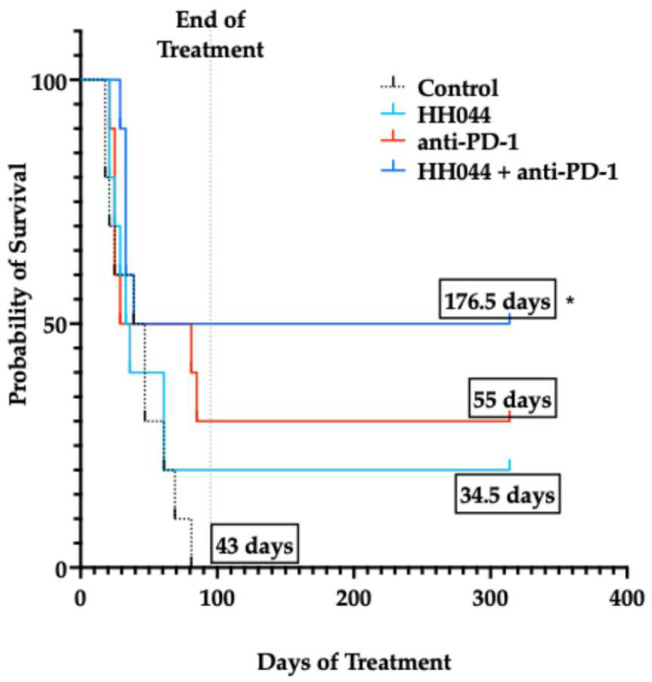
Kaplan–Meier survival curves for experimental treatment groups. Probability of survival in mice treated with HH044, either as monotherapy or in combination with anti-PD-1 immune checkpoint blockade. Median survival of treatment groups (in days) is displayed in black boxes. * *p* < 0.05 compared to the control group.

As shown in Figure 6, by day 81 post-tumor inoculation, all the mice in the control group had reached the survival endpoint. The mice treated with HH044 or MAC-3-190 alone were not found to have significantly different median survival values compared with that of the control group. Anti-PD-1 immunotherapy extended the median survival to 55 days, but the difference was not statistically significant compared with the control (43 days).

Of all the treatment groups, anti-PD-1 in combination with HH044 significantly extended the median survival from 43 days in the control group to 176.5 days (Figure 6, *p* < 0.05), with half of the group being tumor-free up to 230 days after inoculation (5/10). However, the combination treatment with MAC-3-190 and anti-PD-1 did not exhibit a significant impact on mouse survival, despite the potent tumor growth inhibition observed at day 18 (Supplementary Figure S8). Further studies on bioavailability and tumor penetration using more dosing regimens are warranted to better understand the distinct in vivo anti-tumor effects observed between these two lead compounds, which will provide valuable insight for future chemical structure optimization and analog design.

Upon reaching the study endpoint, the mice were euthanized, and organs were collected and weighed. Enlarged cecum were noted in the mice receiving HH044 treatment for ≥21 days. The cecum weights were significantly increased compared with that of the control group (*p* < 0.0001; Supplementary Figure S7b). However, such alterations were only observed during autopsy, and the mice remained active with no behavioral changes or other observable signs of severe toxicity or distress during treatment.

### 3.8. Ex Vivo Susceptibility of Melanoma Cells to nNOS Inhibition After Prolonged Treatment

**Figure 7:**
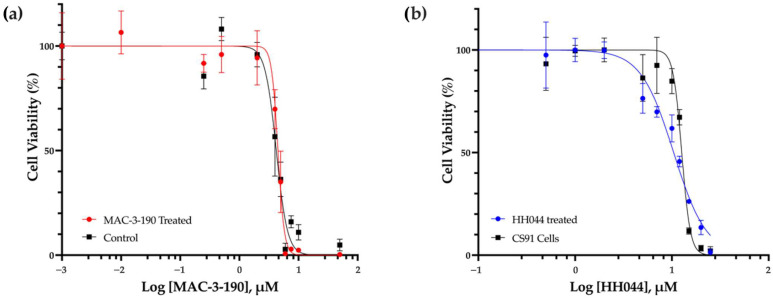
Ex vivo susceptibility of Cloudman S91 cells to nNOS inhibitors. CS91 cells were isolated from tumors growing in male DBA/2 mice treated with intraperitoneal injections of either vehicle control or nNOS inhibitor (10 mg/kg/day). The MAC-3-190 and HH044 IC_50_ values were determined by an MTT colorimetric assay. (**a**) CS91 cells were isolated from the tumors of the control- or MAC-3-190-treated mice. (**b**) CS91 cells were isolated from the tumors of HH044-treated mice in comparison to in vitro cultured CS91 cells.

CS91 cells were isolated from the vehicle control-treated tumors and tumors that initially appeared to respond to MAC-3-190 or HH044 (10 mg/kg/day) treatment but continued growing, reaching ~1500 mm^3^ by measurement. Single-cell suspensions were collected and cultured in growth medium. The cultured CS91 cells were then subjected to MTT analyses to determine whether resistance to nNOS inhibitors had developed after prolonged in vivo treatment. As shown in Figure 7a, the IC_50_ of the CS91 cells isolated from the control-treated mice (4.2 µM) was found to be similar to that of the MAC-3-190-treated mice (4.5 µM). Cells that were isolated from the tumors with HH044 treatment showed similar sensitivity to the in vitro cultured CS91 cells (10.44 μM and 12.65 μM, respectively) (Figure 7b). This suggests that prolonged treatment of nNOS inhibitors did not change the sensitivity to MAC-3-190 or HH044. No development of resistance was evident.

## 4. Discussion

In our study, we investigated the therapeutic potential of combining small-molecule nNOS inhibitors with PD-1 blockade to enhance the anti-melanoma activity. Our mechanistic studies—including transcriptomic analysis, ex vivo PBMCs and melanoma co-culture, and an in vivo immunocompetent mouse melanoma model—demonstrated that blockade of nNOS modulated the T cell immune profile and enhanced T cell activation, thereby influencing anti-tumor immunity. Our results, for the first time, show that nNOS inhibitors in combination with ICIs can be a novel and promising strategy to improve melanoma therapy.

### 4.1. Effect of nNOS Inhibitor Treatment on Oncogenic Signaling Pathways

Our NanoString transcriptomic analysis demonstrated that the Notch, Hedgehog, and Wnt signal pathways were predominantly impacted by HH044 treatment. These pathways play a fundamental role in organismal development and growth, regulating the core cellular processes involved in proliferation, differentiation, and migration [61]. Aberrant activation of these developmental pathways has been shown to play an essential role in the development of resistance to anticancer therapies [62]. BRAF inhibitors have been shown to activate the Sonic Hedgehog Homolog pathway in melanoma, which in turn will upregulate PDGFRα, leading to the development of resistance to the inhibitor [63]. Inhibition of the pathway can in turn increase the sensitivity to BRAF inhibition. Similarly, the development of resistance to BRAF inhibitors in melanoma has been shown to be linked to the upregulation of Notch signaling, while inhibiting this signaling pathway resensitized the cancer to the targeted therapy [64,65,66,67]. Inhibiting Notch1 enhanced immunotherapy efficacy in melanoma [65]. On the other hand, a tumor suppressor role for Notch1 and Notch2 was reported in a BRAF^V600E^/Pten^null^ transgenic model of mouse melanoma [68]. In our study, HH044 downregulated WNT5A, a contributor to Hedgehog signaling and a known druggable target for melanoma, and nNOS inhibition did not have a direct effect on Notch1 or Notch2 expression alone. However, HH044 increased Notch2 expression in the presence of IFN-γ. These results warrant further analysis of the downstream effects of nNOS inhibition on Hedgehog and Notch signaling.

Wnt signaling has been well studied in melanoma transformation, proliferation, and metastasis. However, the exact role of this signaling pathway in the context of melanomagenesis is still highly controversial [69]. WNT5A is a common activator of non-canonical Wnt signaling, while AXIN1 is the central scaffold protein in the destruction complex involved in lowering β-catenin levels in canonical signaling [70,71]. HH044 treatment was shown to decrease WNT5A, likely decreasing the non-canonical signaling responsible for mediating the migration and motility of melanoma cells associated with metastasis [72]. Additionally, AXIN1 expression was increased by HH044, leading to lower β-catenin levels and thus preventing the activation of canonical signaling implicated in increasing the expression of genes such as MITF and APE-1, which protect melanoma from cellular damage from reactive oxygen species [73]. Ongoing research has focused on the development of therapies targeting the Wnt pathway in melanoma, with a few inhibitors showing promising complementary activity to immune checkpoint blockade [74,75]. Recently, an inverse correlation between activation of the Wnt pathway and T cell infiltration was found in metastatic melanoma patients [76,77].

Matrix metalloproteinases (MMPs) are a family of enzymes that play a key role in the migration, invasion, and metastasis of melanoma cells and may have prognostic value in identifying patients at higher risk of melanoma progression [54,78,79]. MMP9 plays a degradative role in the extracellular matrix, which allows for metastatic tissue invasion and blood extravasation by bypassing this physical barrier [80]. It is also implicated in activating VEGFA, further assisting in angiogenesis and catalyzing the proteolytic cleavage of histone H3 N-terminal tail, promoting pro-melanomagenic transcription [55,81]. MMP1 has also been linked to tumorigenesis and metastasis of melanoma by generating activated TGFβ [82,83]. The overexpression of MMP-1 in melanoma cells is driven by constitutive activation of the ERK pathway, attributed to BRAF mutation and autocrine fibroblast growth factor signaling [84]. MMP1 knockdown significantly diminished the metastatic capacity in vivo [85]. Patients with higher serum levels of MMP9 have been correlated with reduced overall survival, and patients with elevated MMP1 had more rapid disease progression [78]. Our study highlighted a decrease in MMP9 and MMP1 at the RNA level after HH044 treatment in the presence or absence of IFN-γ, complementing the promising anti-tumorigenic activity of our novel nNOS inhibitors.

### 4.2. nNOS Inhibition Enhanced the Anti-Tumor Activity of Immunotherapy

Despite the encouraging response in a subset of patients, only approximately 33–44% of patients respond to anti-PD-1 therapy [86]. Of the patients that initially respond to ICIs, some later develop resistance to the therapy. Many immune resistance mechanisms have been studied that contribute to disease progression, such as T cell tolerance to antigens, T cell exhaustion, checkpoint inhibition, tumor escape from immune surveillance, and increased expression of immune checkpoint ligands [87,88]. Melanoma is highly immunogenic. However, melanoma cells can escape from immune surveillance through the selection of tumor cells resistant to immune-mediated elimination [89,90]. In addition, exhausted T cells exhibit decreased effector functions due to chronic antigen stimulation, which gives rise to the expression of inhibitory receptors, preventing persistent T-cell receptor (TCR) stimulation and subsequent cell death [91]. Moreover, due to the risk of severe irAEs, unpredictable patient response to ICIs, and significant cost, identifying alternative treatment options to enhance anticancer immunotherapy is vital and highly impactful.

Our study showed that nNOS inhibitor monotherapy effectively inhibited tumor growth to similar rates as anti-PD-1 treatment. When used in combination with PD-1 blockade, both HH044 and MAC-3-190 further inhibited tumor growth and exhibited significant tumor volume reduction compared with the control group, with a subsequent increase in survival. Previous studies performed by our group have determined that novel nNOS inhibitors effectively decrease IFN-γ-induced PD-L1 expression in human melanoma cells both in vitro and in vivo [17]. The downregulation of PD-L1 induced by the nNOS inhibitor itself may contribute to the promising in vivo activity seen when combined with anti-PD-1, essentially blocking the PD-1 and PD-L1 immune checkpoint from both sides.

As indicated by MTT analysis and previous in vivo studies with an immune-deficient nude mouse model [17], nNOS inhibitors exhibit direct cytotoxicity to melanoma cells independent of PD-1- and PD-L1-mediated immune suppression. After prolonged treatment, ex vivo cultured tumor cells from MAC-3-190- or HH044-treated mice remain susceptible to nNOS inhibitors. No significant drug resistance was observed in our study. Consequently, targeting nNOS not only effectively reduced melanoma cell viability but also interfered with PD-L1-mediated tumor immunosuppression. As a result, nNOS inhibitors enhanced the anti-melanoma activity of ICIs, which has great translational potential as a novel and promising treatment for patients with melanoma.

NO is also involved in regulating muscle tone in the sphincter of the lower esophagus, pylorus, sphincter of Oddi, and anus [92]. Gastrointestinal changes were reported in animal studies after nNOS inhibitor treatment, with delayed gastric emptying and colonic transit [93]. In a recent study, antibiotics were found to alter the expression of nNOS in the murine gut, resulting in similar observations along with an increase in the thickness of muscularis externa in the stomach, ileum, and cecum [94]. Enlarged stomachs with hypertrophy of the pyloric sphincter were also observed in transgenic mice with homozygous depletion of the nNOS gene [95]. In our study, the mice treated with HH044 either alone or in combination with anti-PD-1 exhibited lower body weights and enlarged ceca at 21 days of treatment compared with the control. The reduced body weight observed in mice with the HH044 treatment (88% of the control) could be the result of reduced gastrointestinal motility. Aside from the reduced body weight and gastrointestinal adverse effects that were only observed during autopsy, the mice remained active with no behavioral changes or other observable signs of severe toxicity or distress.

Moreover, in a preliminary study where HH044 was administered via oral gavage (Supplementary Figure S7c), we failed to observe any significant differences in cecum weight at any of the HH044 doses (10 mg/kg, 25 mg/kg, and 50 mg/kg, per os daily). This suggests that the enlarged cecum might be, at least partially, due to local exposure when administered via intraperitoneal injection. Future studies on bioavailability and biodistribution are warranted to better understand the distinct gastrointestinal toxicities observed between these two lead compounds.

### 4.3. Effect of nNOS Inhibitors on T Cell Activity and Immunophenotypic Changes

IL-2, approved by the FDA in 1998, is a commonly used cytokine serving as adjuvant treatment for patients with melanoma. It has been shown to enhance anti-tumor immunity by promoting the proliferation and activation of cytotoxic T lymphocytes and natural killer (NK) cells, thereby augmenting the immune system’s ability to target and eliminate malignant cells [96]. As such, IL-2 positivity was utilized as a marker for T cell activation in the human melanoma and T-cell co-culture studies. Our results demonstrate that nNOS inhibitor treatment prevented T cells from melanoma-induced immunosuppression, as indicated by significant increases in IL-2^+^ cells in the CD3^+^ and CD4^+^ T cell populations. Consistently, our previous study showed that co-treatment with the nNOS inhibitor effectively decreased PD-L1 staining in murine tumor tissues compared with the IFN-γ-treated mice [17]. Thus, T cell activation indicated by increased IL-2^+^ cells might be attributed to the inhibition of PD-L1 expression in melanoma cells during co-incubation. An earlier study reported by Liu et al. showed that elevated nNOS expression in human melanoma tissue was linked to immune dysfunction of circulating T cells, resulting in immunosuppression [18]. Although further mechanistic studies are warranted, this observational study, in combination with our studies, revealed a critical role of nNOS-mediated NO signaling in regulating the immune responses of human melanoma within the tumor microenvironment [16,17].

The significant increase in IL-2^+^ cells was found primarily in CD4^+^ cells, which may increase the expression level of antigen presentation machinery and costimulatory molecules by antigen-presenting cells (APCs), facilitating the priming, expansion, memory formation, and survival of CD8^+^ cytotoxic T cells [97]. We further determined if certain T cell subsets at baseline correlated with donors’ responses to nNOS inhibitor pretreatment. Seven donors were classified as responders, while four were identified as non-responders. Human PBMC-derived T cell phenotyping revealed that the percentage of CD4^+^ naïve and CD4^+^RORγt^+^ cells were significantly different between the responding and non-responding donors. CD4^+^ T cells require appropriate activation in vivo for tumor clearance, as naïve CD4^+^ T cells cannot differentiate into tumor-specific CD4 effector cells [98]. Abundant naïve CD4 cells were shown to be associated with poor patient prognosis and upregulated T_regs_ in breast tumors [99]. This is consistent with our observations that donors with more naïve T cells were not responsive to nNOS inhibitor treatment.

RORγt is a transcription factor expressed in Th17 cells and plays a critical role in the secretion of IL-17. Studies have shown that Th17 cells infiltrate the tumor microenvironment in melanoma, ovarian cancer, and colon cancer, suggesting that increased Th17 infiltration may play a role in cancer pathogenesis [100]. Tumor-specific Th17 cells have been shown to eradicate advanced melanoma in an adoptive transfer therapeutic tumor model [101]. These cells can also further differentiate into Th17 and Th1 CD4 cells, which secrete IL-2 [102]. Our analysis showed that the responding donors exhibited a greater percentage of CD4^+^RORγt^+^ T cells at baseline before co-culturing with melanoma cells. RORγt, co-expressed with FOXP3, maintains immunological homeostasis and immune tolerance, differentiating these cells into regulatory T cells (T_reg_) [103]. However, we did not observe any significant difference in T_reg_ populations between the responding and non-responding donors. Our data suggests that naïve CD4^low^ and CD4^+^RORγt^hi^ cells at baseline may be potential biomarkers to predict a patient’s response to the combination therapy with nNOS inhibitors and ICIs.

Moreover, multiple factors influence whether a patient will respond to immunotherapy. PD-L1 expression remains a long-debated biomarker, and current recommendations do not limit ICI use to patients with high PD-L1 expression [104]. Earlier clinical studies have shown that patients who express less than 1% PD-L1 may have improved progression-free survival from a combination anti-PD-1 and anti-CTLA-4 therapy [105]. Simon et al. defined an active tumor microenvironment as having an accumulation of CD8^+^PD-1^+^ activated effector T cells, indicating a sub-population of about 38% of cancer patients that are able to respond to anti-PD-1 therapy [106]. The presence of activated CD8^+^PD-1^+^ T cells in patient blood was also associated with therapeutic efficacy. Conversely, the CD8^+^PD-1^+^ T cells we observed could be representative of an exhausted population, and our current analysis panel lacks the markers to distinguish exhausted cells from PD-1-expressing memory populations [107,108]. However, our in vivo study showed a significant increase in CD8^+^PD-1^+^ cells in the periphery after treatment with HH044 in comparison with the control mice, and the combination therapy with HH044 and anti-PD-1 had better antitumor activity than each treatment alone, which suggests to us that nNOS inhibition enhances the effect of ICI therapy in this model. Based on our findings, this study would support the conclusion that the PD-1^+^ T cells are capable of functional reactivation in this context and are more likely to be functionally inhibited memory populations rather than exhausted populations.

## 5. Conclusions

In summary, our study shows promise in developing first-in-class nNOS inhibitors for melanoma therapy, either alone or in combination with immune checkpoint blockade. Our innovative double-sided approach targeting both nNOS-stimulated melanoma progression and PD-L1-mediated tumor immunosuppression effectively enhanced the anti-melanoma activity of immunotherapy without producing more severe toxicity. The use of nNOS inhibitors for treating melanoma patients is highly promising and holds significant translational potential.

## Figures and Tables

**Table 1:** Novel selective nNOS inhibitors.

Compound	*K*_i_ (μM)				Selectivity			Cytotoxicity [17]
	Rat nNOS (rn)	Human nNOS (hn)	Human iNOS (hi)	Human eNOS (he)	hn/rn	hn/hi	hn/he	IC_50_ (μM)
HH044	0.005	0.02	1.215	6.735	4	61	337	5.27 ± 3.3
MAC-3-190 *	0.033	0.051	4.54 (murine)	6.09	1.5	89 (hn/mi)	119	1.21 ± 0.19

hn = human nNOS; rn = rat nNOS; hi = human iNOS; he = human eNOS; mi = mouse iNOS; * Compound **14** [47].

## Data Availability

The original contributions presented in this study are included in the article or Supplementary Materials. Further inquiries can be directed toward the corresponding authors.

## Supplementary Materials

The following supporting information can be downloaded at https://www.mdpi.com/article/10.3390/pharmaceutics17060691/s1. Table S1: Significantly upregulated and downregulated genes after treatment with HH044 in comparison to control related to significantly affected signaling pathways. Table S2: Significantly upregulated and downregulated genes after cotreatment with IFN-γ in comparison to control related to significantly affected signaling pathways. Table S3: Significantly upregulated and downregulated genes after cotreatment with HH044 and IFN-γ in comparison to IFN-γ alone related to significantly affected signaling pathways. Figure S1: Flow cytometry gating strategy to identify mouse CD3+, CD4+, CD8+, PD-1+, GATA3+, Tbet+, RORγT+, FOXP3+, and RORγT+ FOXP3+ double-positive T cell populations. Figure S2: Flow cytometry gating strategy to identify human CD3+, CD4+, CD8+, and IL-2+ T cell populations. Figure S3: Contour plots of changes in IL-2+ cells shown in Figure 3a. Figure S4: Changes in IL-2+ T cell frequencies after treatment with IFN-γ in combination with nNOS inhibitors. Figure S5: Contour plots of changes in IL-2+ cell treatment with nNOS inhibitors with and without IFN-γ in Donor 03. Figure S6: Flow cytometry gating strategy for T cell immunophenotyping panel. Figure S7: Body and cecum weights of mice treated with nNOS inhibitors. Figure S8: Kaplan–Meier survival curves for experimental treatment groups.

## Abbreviations

The following abbreviations are used in this manuscript:

ANOVA	Analysis of variance
APC	Antigen-presenting cell
CM	Cutaneous melanoma
COX-2	Cyclooxygenase 2
CS91	Cloudman S91
CTLA-4	Cytotoxic T-lymphocyte associated protein 4
IACUC	Institutional Animal Care and Use Committee
ICI	Immune checkpoint inhibitors
IFN-γ	Interferon-gamma
IL-2	Interleukin-2
irAE	Immune-related adverse event
MMP	Matrix metalloproteinase
nNOS	Neuronal nitric oxide synthase
NO	Nitric oxide
ORR	Overall response rate
OS	Overall survival
PBMC	Peripheral blood mononuclear cell
PD-1	Programmed cell death protein 1
PD-L1	Programmed cell death protein ligand 1
PGE2	Prostaglandin E2
PMA	Phorbol 12-myristate 13-acetate
SEM	Standard error of the mean
Treg	Regulatory T cell
