# Supplementary material for: Targeting Neuronal Nitric Oxide Synthase (nNOS) as a Novel Approach to Enhancing the Anti-Melanoma Activity of Immune Checkpoint Inhibitors
Source: Pharmaceutics. 2025 May 24;17(6):691. doi: 10.3390/pharmaceutics17060691 (PMC12196278; doi:10.3390/pharmaceutics17060691)

Table S1: Significantly upregulated and downregulated genes after treatment with HH044 in comparison to control related to significantly affected signaling pathways.

Pathway	Significance Score	Downregulated genes	Upregulated genes
Wnt signaling	2.2673	WNT5A	AXIN1
Hedgehog signaling	2.1423	WNT5A, BMP2	IHH
Matrix remodeling and metastasis	1.94	PDGFB, MMP9, LAMB3, ITGAV, LAMC2, ITGA1, ITGB8, PECAM1, THBS1, BMP2	LTBP1
TGF-beta signaling	1.8043	BMP2, ID4, THBS1	LTBP1
Angiogenesis	1.7702	EDN1, TYMP, PDGFB, THBS1, ITGAV, MMP9	VEGFA, NFIL3
NF-kappaB signaling	1.6256	TNFRSF25, CD40, TNFRSF14	
Lymphoid compartment	1.6172	TNFRSF25, ITGA1, CD40	EOMES
Immune cell adhesion and migration	1.5861	PECAM1, ITGA1, ITGB8, MMP9, CD40, HLA-DRB5, ITGAV	-
P13K-Akt	1.5824	PDGFB, THBS1, CDK6, ITGAV, LAMC2, LAMB3, GNG4, ITGB8, ITGA1	EGF, VEGFA
Hypoxia	1.5394	EDN1	HK2, EGF, PFKFB3, VEGFA

Table S2: Significantly upregulated and downregulated genes after cotreatment with IFN-γ in comparison to control related to significantly affected signaling pathways.

Pathway	Significance Score	Downregulated genes	Upregulated genes
TGF-beta signaling	2.6625	ID4, BMP2, INHBA	MYC, LTBP1, TGFBR1
Matrix remodeling and metastasis	2.4971	MMP1, LAMB3, ITGB8, ITGA1, LAMC2, NCAM1, BMP2, ITGAV, ITGA2	NID2, ITGA4, LTBP1, SPP1, COL6A3
Hedgehog signaling	2.4318	BMP2, WTN5A	PSMB8
Immune cell adhesion and migration	2.1476	CXCR4, ITGAV, NCAM1, ITGB8, ITGA2	HLA-F, HLA-DPA1, HLA-A, ITGA4, CD8A, HLA-DRA
NF-kappaB signaling	2.0558	BIRC3, NFKB1, TNFRSF25, NFKB2, RELB	PSMB8
Lymphoid compartment	2.0212	TNFRSF25, ITGA1, IL11, TBX21	DPP4, CD8A, IFITM1, HLA-DOB, F2RL1, GZMK
Antigen presentation	2.0196	VHL, ITGAV	CD8A, HLA-B, HLA-DPB1, HLA-E, HLA-DOB, HLA-DPA1, B2M, PSMB8, TAP2, HLA-F, HLA-DRA, HLA-A
Interferon signaling	2.0155	IRF7, NCAM1	GBP4, HLA-F, GHR, HLA-A, PSMB8, IFITM1, HLA-DPA1, HLA-DPB1, HLA-E, IRF5, HLA-B, HLA-DRA, B2M, IFIT2
P13K-Akt	1.9989	ITGB8, CDKN1A, ITGAV, LAMC2, LAMB3, FGFR1, NFKB1, ITGA2, ITGA1	MYC, GHR, ITGA4, SPP1, COL6A3, FGF13
Hypoxia	1.9915	CDKN1A, VHL, NFKB1	PFKFB3, HK2

Table S3: Significantly upregulated and downregulated genes after cotreatment with HH044 and IFN-γ in comparison to IFN-γ alone related to significantly affected signaling pathways.

Pathway	Significance Score	Downregulated genes	Upregulated genes
Notch signaling	1.7019	-	MYC, TP53, NOTCH2
Matrix remodeling and metastasis	1.6136	MMP1, A2M, NCAM1	NID2, COL6A3, ITGA4
P13K-Akt	1.6044	CDKN1A, PRKAA2, PTEN	FLT1, BAD, COL6A3, PDGFRB, GNG4, TP53, MYC, ITGA4, FGF13
Autophagy	1.5853	PRKAA2, PTEN	BAD
Hypoxia	1.5236	CDKN1A	FLT1
MAPK	1.4797	-	FLT1, GNG4, TP53, MYC, FGF13, PDGFRB, BAD
Cell proliferation	1.4753	CDKN1A	CCNB1, TP53, MYC
Metabolic stress	1.4603	PRKAA2, CDKN1A, PTEN	MYC, TP53, PDGFRB
JAK-STAT signaling	1.4508	IL24, CDKN1A, SOCS1	MYC
Cytokine and chemokine signaling	1.426	CXCR4, IL16, CCL3/L1, IL24	GNG4

Figure S1: Flow cytometry gating strategy to identify mouse CD3$^+$, CD4$^+$, CD8$^+$, PD-1$^+$, GATA3$^+$, Tbet$^+$, RORγT$^+$, FOXP3$^+$, and RORγT$^+$ FOXP3$^+$ double positive T cell populations.

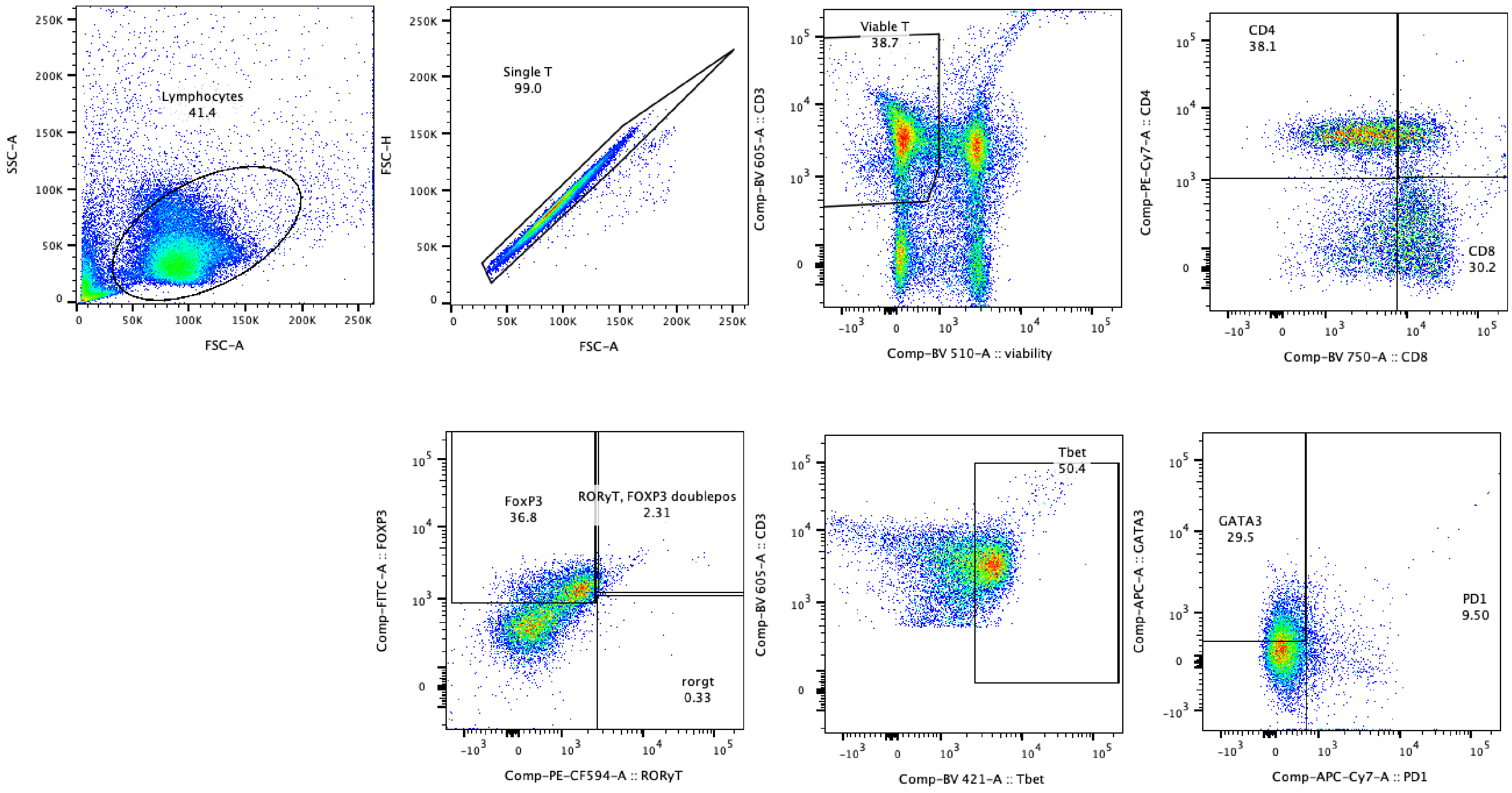

Figure S2: Flow cytometry gating strategy to identify human CD3⁺, CD4⁺, CD8⁺, and IL-2⁺ T cell populations.

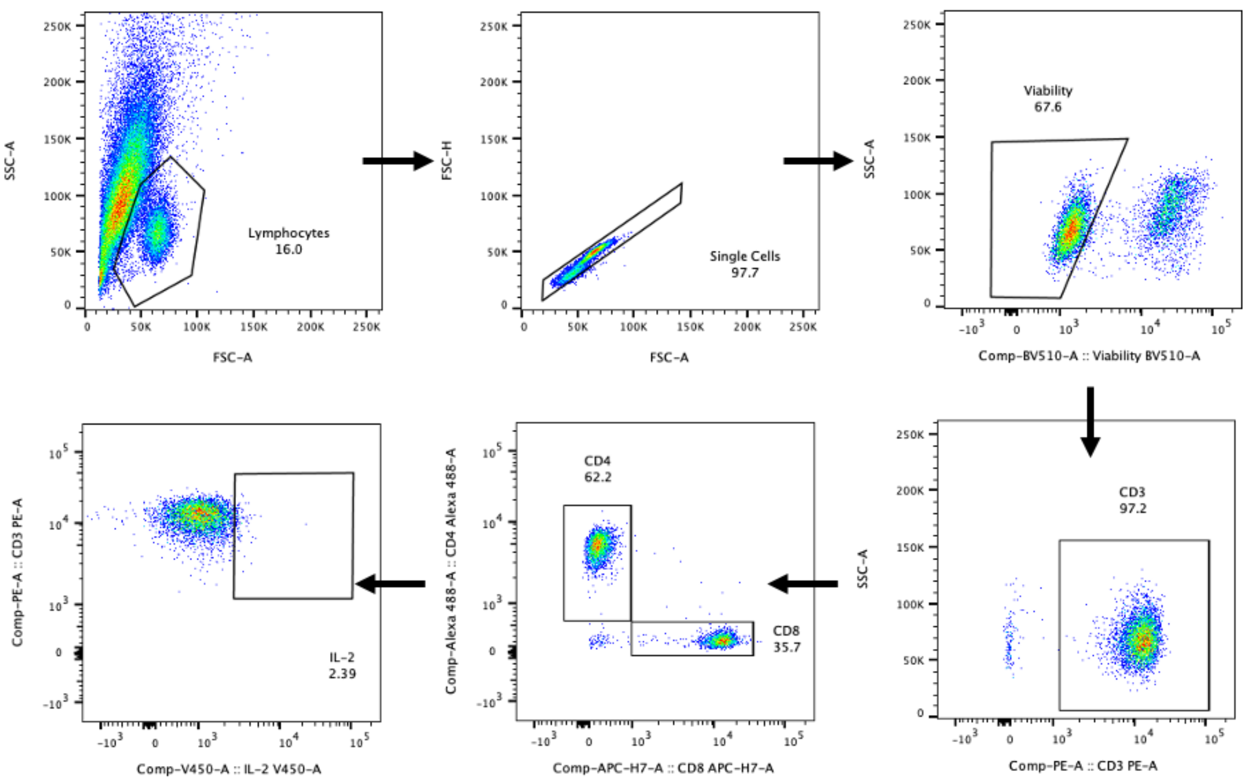

Figure S3: Contour plots of changes in IL-2$^+$ cells shown in Figure 3a.

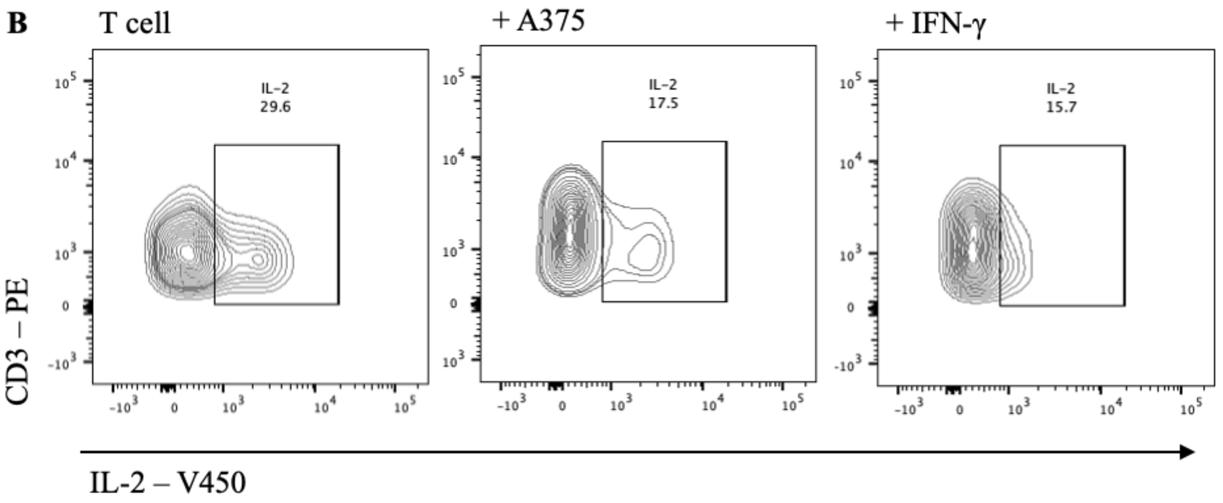

Figure S4: Changes in IL-2$^+$ T cell frequencies after treatment with IFN-γ in combination with nNOS inhibitors. CD3$^+$IL-2$^+$, CD4$^+$IL-2$^+$, and CD8$^+$IL-2$^+$ T cells slightly increased after co-treatment of 250 U/mL IFN-γ and 3.0 µM MAC-3-190 compared to T cells co-incubated with IFN-γ-treated A375 (n=11).

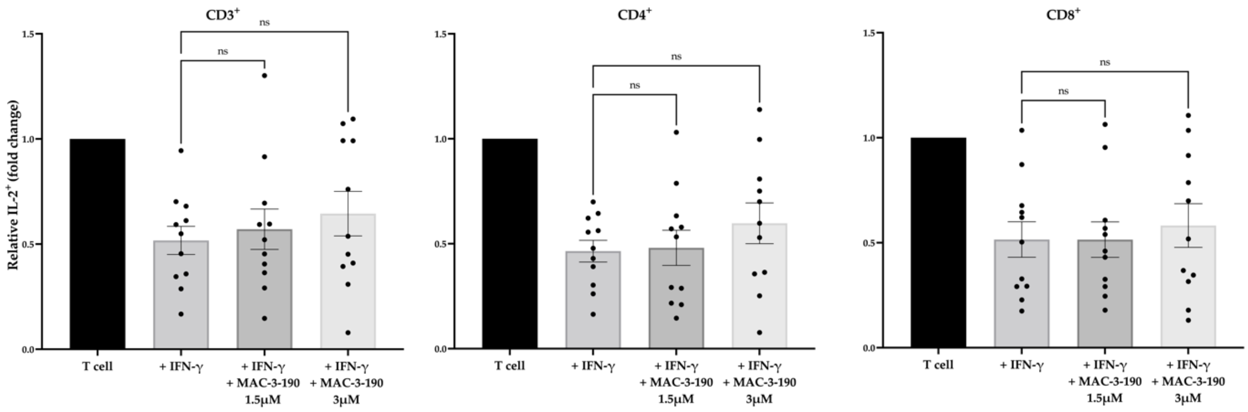

Figure S5: Contour plots of changes in IL-2+ cell treatment with nNOS inhibitors with and without IFN-γ in Donor 03.

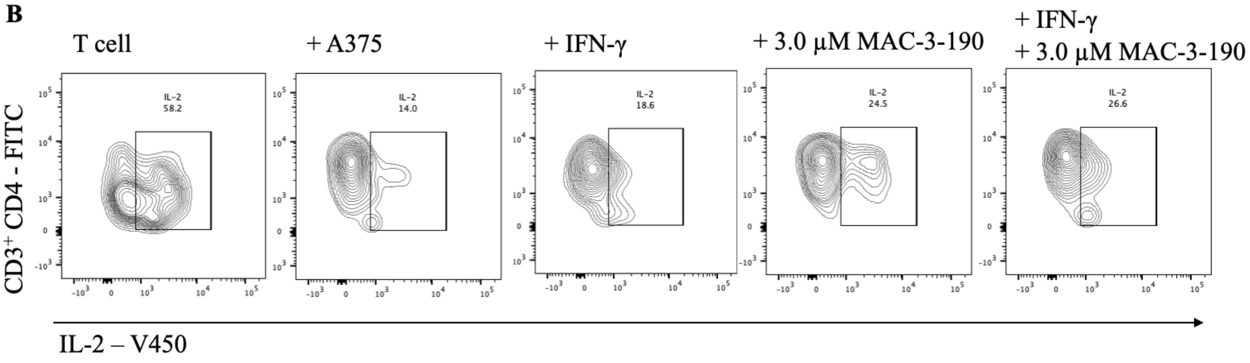

Figure S6: Flow cytometry gating strategy for T cell immunophenotyping panel.

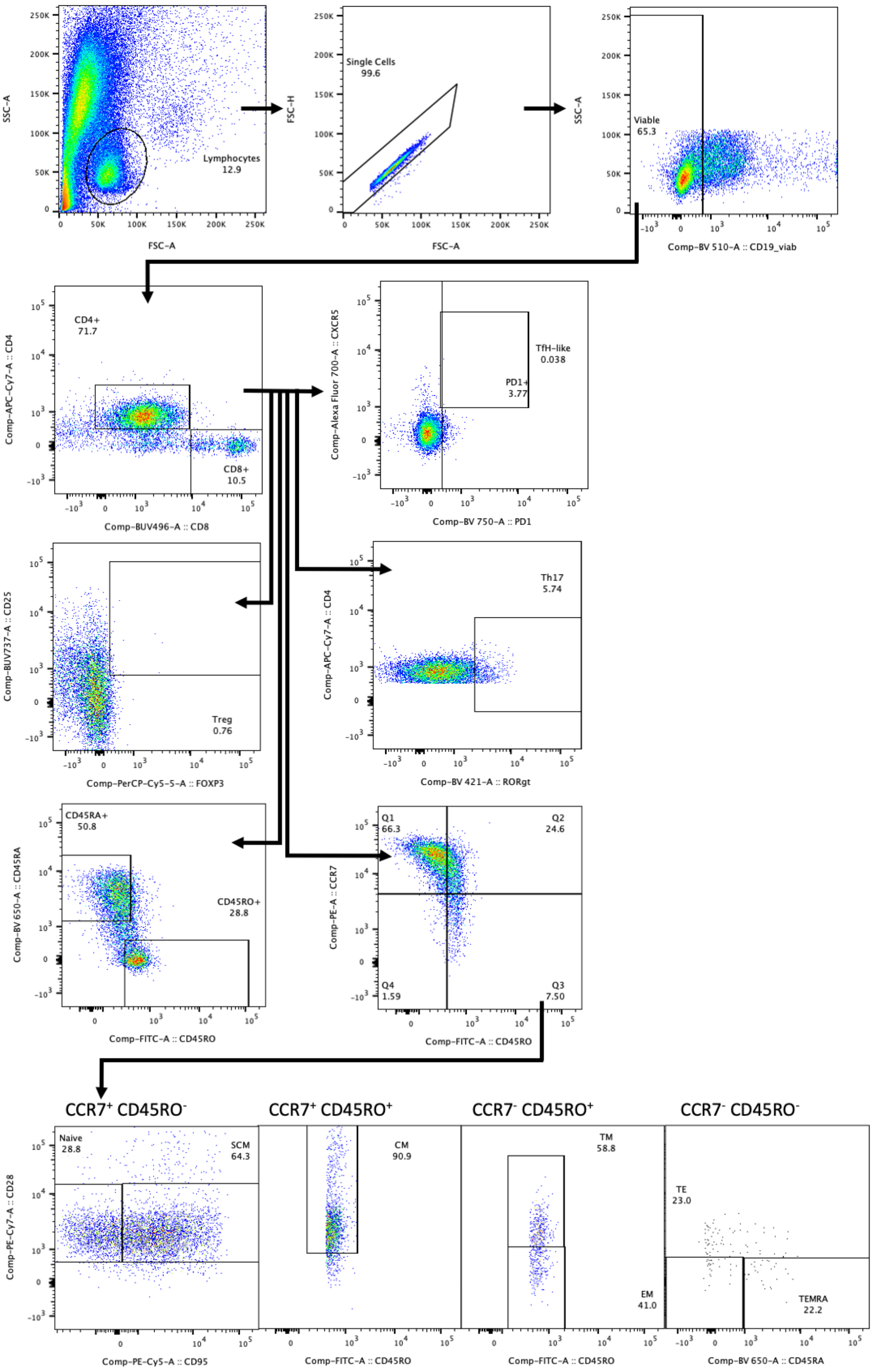

Figure S7. Body and cecum weights of mice treated with nNOS inhibitors. Mice were treated with either HH044 or MAC-3-190 (10 mg/kg) alone or in combination with anti-PD-1 or anti-CTLA-4 immunotherapy. **a)** Body weights of mice treated with HH044 were significantly lower than control on day 21 (**, p < 0.005; ***, *p* < 0.0005). **b)** Cecum weights of mice treated for more than 21 days. All groups treated with HH044 exhibited significant cecum enlargement (***, *p* < 0.0001, compared to control). Significance was determined using ordinary one-way ANOVA analysis. **c)** Cecum weight of mice treated with HH044 (10mg/kg/day, 25mg/kg/day, and 50mg/kg/day) administered via oral gavage.

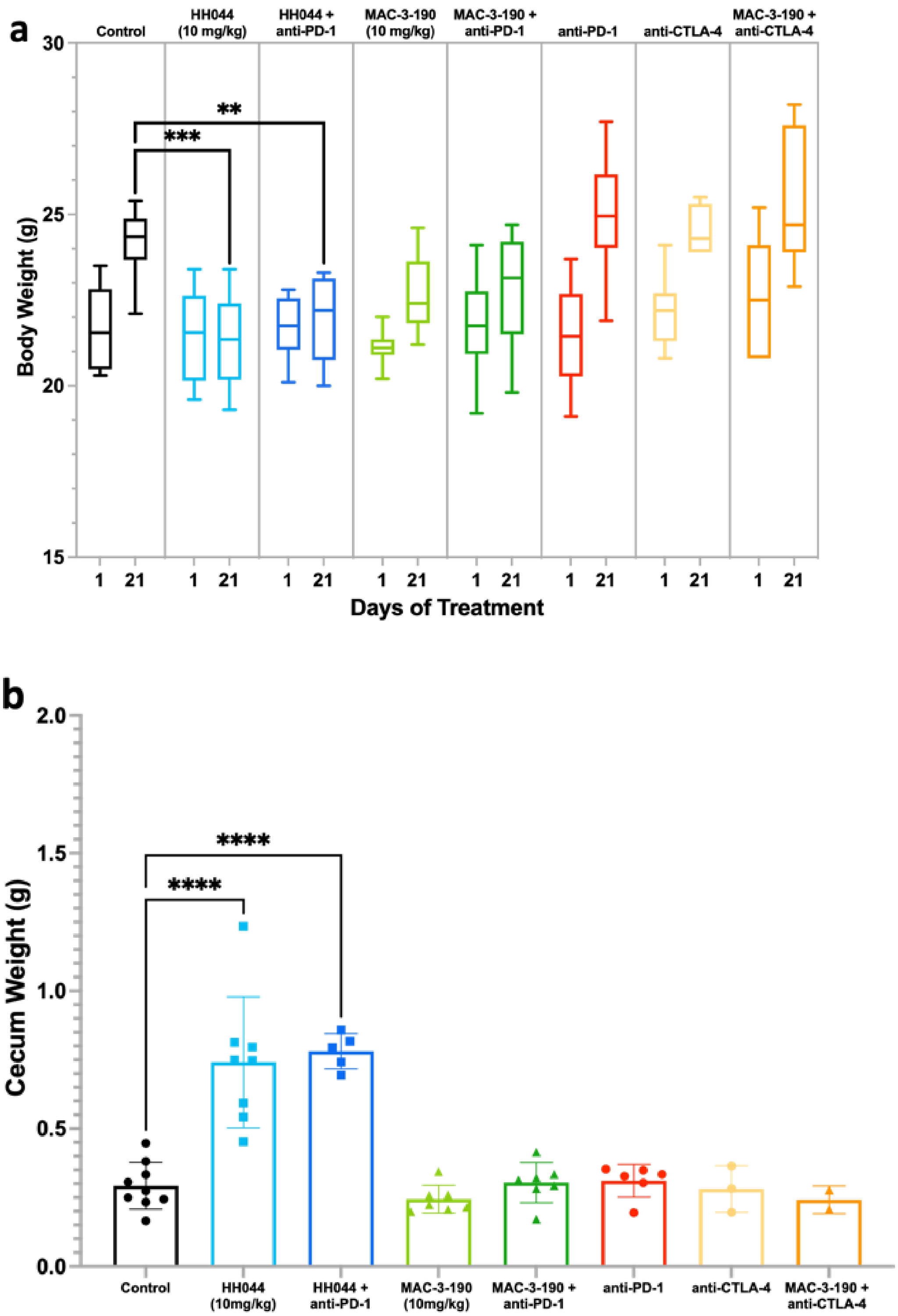

c

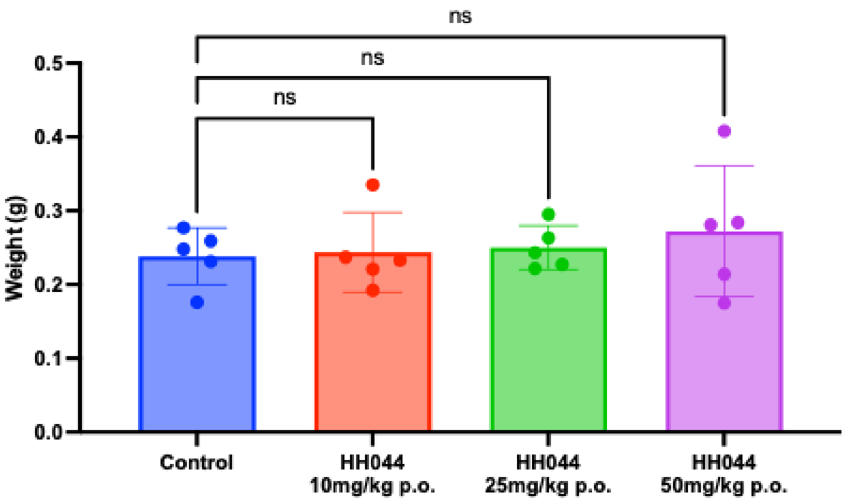
ns
ns
ns
0.5
0.4
0.3
0.2
0.1
0.0
Weight (g)
Control
HH044
10mg/kg p.o.
HH044
25mg/kg p.o.
HH044
50mg/kg p.o.

Figure S8: Kaplan-Meier survival curves for experimental treatment groups. Probability of survival in mice treated with MAC-3-190 either as monotherapy or in combination with anti-PD-1 immune checkpoint blockade. Median survival of treatment groups in days are displayed in black boxes.

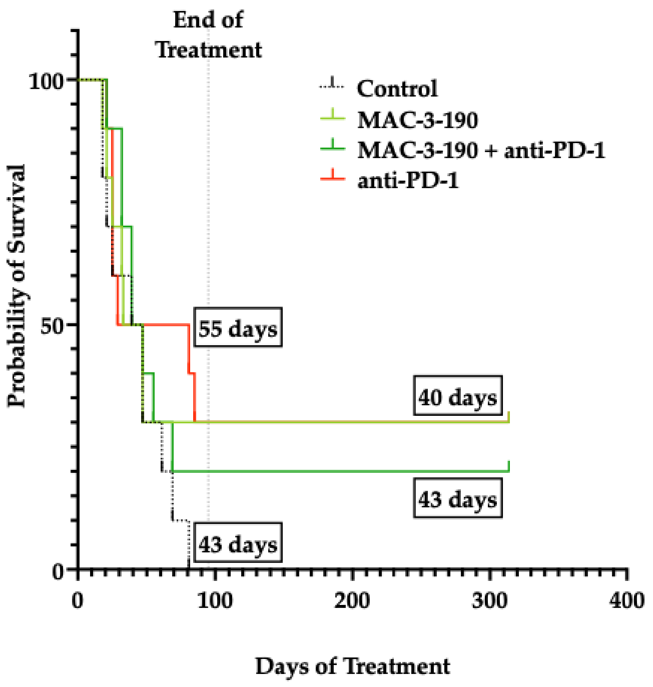

Supplement: Supplementary file 1 [file pharmaceutics-17-00691-s001.zip › pharmaceutics-3605304-supplementary.pdf]
